# Recent Advances in Tissue Adhesives for Clinical Medicine

**DOI:** 10.3390/polym12040939

**Published:** 2020-04-18

**Authors:** Liangpeng Ge, Shixuan Chen

**Affiliations:** 1 Chongqing Academy of Animal Sciences and Key Laboratory of Pig Industry Sciences, Ministry of Agriculture, Chongqing 402460, China; 2 Department of Surgery-Transplant and Mary & Dick Holland Regenerative Medicine Program, University of Nebraska Medical Center, Omaha, NE 68105, USA

**Keywords:** tissue adhesives, natural adhesives, synthetic adhesives, biomimetic adhesives, applications

## Abstract

Tissue adhesives have attracted more attention to the applications of non-invasive wound closure. The purpose of this review article is to summarize the recent progress of developing tissue adhesives, which may inspire researchers to develop more outstanding tissue adhesives. It begins with a brief introduction to the emerging potential use of tissue adhesives in the clinic. Next, several critical mechanisms for adhesion are discussed, including van der Waals forces, capillary forces, hydrogen bonding, static electric forces, and chemical bonds. This article further details the measurement methods of adhesion and highlights the different types of adhesive, including natural or biological, synthetic and semisynthetic, and biomimetic adhesives. Finally, this review article concludes with remarks on the challenges and future directions for design, fabrication, and application of tissue adhesives in the clinic. This review article has promising potential to provide novel creative design principles for the generation of future tissue adhesives.

## 1. Introduction

Tissue adhesives are increasingly important elements in modern medicine, with rapid development over the past 30 years. They enable the innate wound healing processes to occur through the adhesion of tissue to the tissue on-site or tissue to non-tissue surfaces on-site, thus exhibiting some attractive characteristics in the clinic, such as less traumatic closure and suffering, easy application, no stitches required after surgery, excellent cosmetic result, and localized drug release [1,2,3,4,5,6]. 

After several decades of intensive research activities, a wide variety of tissue adhesives have been developed to increase the ability of tissue adhesives to meet different clinic requirements. The current clinic used tissue adhesives could be divided into three groups: (1) natural tissue adhesives, (2) synthetic and semisynthetic tissue adhesive, and (3) biomimetic tissue adhesives [2]. These tissue adhesives are extensively used different tissues, including skin [7,8,9], breast [10,11], cardiac [12,13,14], gastrointestinal [15], head and neck [16,17], hepatic [18,19], neurological [20], orthopedic [9,21], pediatric [8,22], thoracic [14,23], bone [24], dental [25] and vascular surgery [26,27]. For example, Dana et al. reported a gelatin-methacryloyl based tissue adhesive (GelCORE) in the presence of a type 2 initiator Eosin Y (initiator), triethanolamine (TEA) (co-initiator), and N-vinylcaprolactam (VC) (co-monomer) for the sutureless repair of corneal injuries. The GelCORE was able to effectively seal the defects of corneal, and promote the regeneration and re-epithelialization of stromal. Gao et al. developed milk casein and polyacrylamide (PAAm) based tissue adhesives, the maximum peeling force could reach to 378 N/m, and the fracture stress was 180 kPa at fracture strain of 2000%. The casein-PAAm tissue adhesive exhibited an outstanding adhesion property on tissues, including liver, heart, lung, spleen, kidney, and bone. Moreover, there were no inflammatory responses after application. Despite the tremendous efforts of the scientific community over the past few decades, currently, commercialized tissue adhesives still have some disadvantages.

Therefore, the objective of this review article is to gather recent information of tissue adhesives, analyze the mechanism of adhesives, describe the specific properties of different kinds of tissue adhesives, discuss the advantages and disadvantages related to their clinical applications, and discover inspiration for these future research efforts to develop the next generation tissue adhesives.

## 2. The Mechanisms of Adhesion

The main adhesion mechanisms of these tissue adhesives include molecular bonding, mechanical coupling, and thermodynamic adhesion [28]. Among them, molecular bonding is the most popular explanation. In brief, interatomic and/or intermolecular forces are established between the molecules at the surface of the tissue and the molecules of adhesive, which are contributions from hydrogen bonding, capillary forces, van der Waals forces, static electric force, and covalent bonds [29].

### 2.1. Van der Waals Force

The van der Waals forces are usually attractive. They exist in any polar or nonpolar molecule and arise from a temporary dipole moment produced by the instantaneous positions of electrons in a molecule. The van der Waals forces are the smallest among all intermolecular forces, but they become significant when a large number of particles are involved at a suitable (nanoscale) distance [30,31,32]. A typical value of van der Waals forces for a sub-micrometer object is ≈100 nN, and this force becomes dominant in the case of hydrophobic surfaces. The van der Waals forces have been found in a serious of other land animals, including geckos [33], tree frogs [34,35], insects [32], and abalone [36]. 

Autumn et al. indicated that van der Waals forces are the dominant adhesive force in geckos’ adhesion in the dry, hairy system [33]. They found that millions of nanoscaled fibrils on the geckos’ feet act together to create a formidable adhesion of ≈10 N cm^2^, sufficient to keep geckos firmly on their feet, even when upside down on a glass ceiling [33,37]. Inspired by geckos, Geim successfully microfabricated a large (1 cm^2^) sample of polyimide pillars mimicking gecko foot-hair, and the results showed that the average force per hair was found to be ≈70 nN, and the whole 1 cm^2^ patch was able to support 3 N [38].

### 2.2. Capillary Forces

Capillary force exists everywhere under natural conditions. Capillary force is also termed meniscus force and usually dominates over other surface forces in the nanoscale contact under a wet adhesion. It is caused by a liquid meniscus around the contact areas of two lyophilic solid surfaces or capillary bridges of one liquid in another immiscible liquid [39]. Capillary forces can significantly contribute to the adhesion of biological and artificial micro- and nanoscale objects. In micromanipulation, the influence of the gravitational force is extremely small compared to the adhesion force. The capillary force is large enough to detach an adhered particle if the liquid has volume sufficiently smaller than that of the particle. 

In the case of hydrophilic materials, capillary forces have been verified as responsible for the adhesion of geckos [37,40,41]. Moreover, animals such as beetles [42], frogs [43], blowflies [44], and ants [45] can also adhere to surfaces by capillary forces through producing a liquid film between the pad and substrate. Lin et al. showed that capillary forces of nanofibrils may contribute to the strong adhesion exhibited by abalone under humidity, and they think future synthetic nanofibril attachment devices can be inspired by this phenomenon. Thus, this broadens its application, including the use of capillary forces in wet environments [36].

### 2.3. Hydrogen Bonding

A hydrogen bond is an interaction in which a hydrogen atom bridges two electronegative atoms (in biological systems, this is usually nitrogen or oxygen). Hydrogen bond strengths can vary from 5 kJ/mole to hundreds of kJ/mole, depending on the molecules and whether they are in a gas or a solution. For example, a guanine–cytosine hydrogen bond has a strength of approximately 80–100 kJ/mole in the gas phase and less than 20 kJ/mole in an aqueous solution [46].

Hydrogen bonding has been widely used for developing self-healing polymers by incorporating strong and reversible noncovalent hydrogen bonding moieties into the polymer structure. For example, Sijbesma et al. first used the 2-ureido-4[1H]-pyrimidinone (UPy) group (a strong quadruple hydrogen bonding dimer) to synthesize supramolecular polymers, and it was highly thermally responsive [47]. Faghihnejad et al. also successfully fabricated self-healing poly (butyl acrylate) (PBA) copolymers containing comonomers with UPy and found that the adhesion strengths of the PBA–UPy polymers depend on the UPy content, due to the strong UPy–UPy H-bonding interactions [48].

**Figure 1:**
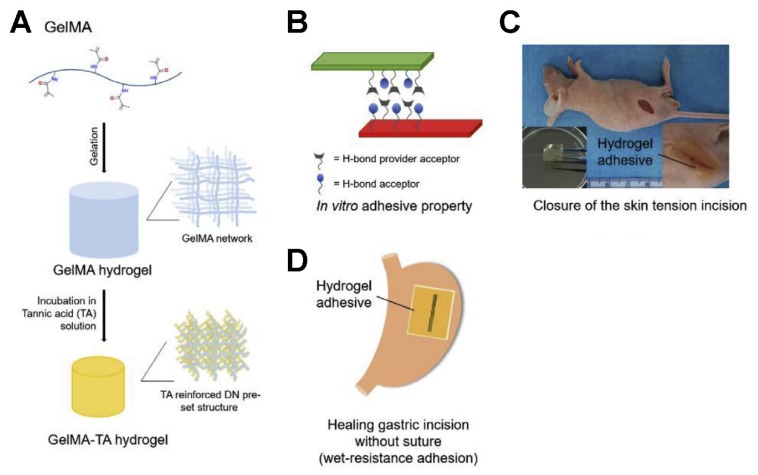
The schematic illustration of preparing multifunctional GelMA-TA hydrogel with high stiffness, super-elasticity, deformability (**A**), and in vivo self-healing and adhesive property (**B**). Biomedical applications of GelMA-TA gel for skin wound closure (**C**), sutureless gastric surgery (**D**). ^49^ Copyright 2018, Elsevier.

Tannic acid (TA) is a natural polyphenol with multiple hydroxyl groups, which provide rich hydrogen bonds. Xing et al. developed a hybrid GelMA (Gelatin-Methacryloyl) hydrogel by incorporating tannic acid (Figure 1). The structural reinforced features of this hybrid hydrogel are contributed by the intermolecular hydrogen bonding of TA, which is simple but strong. This kind of GelMA-TA hydrogel with an adjustable proportion of components can achieve superior mechanical properties. The low concentration of GelMA is conducive to achieve the highest deformability. Also, a high concentration of TA cannot only enhance the mechanical property of the hydrogel but also increase the adhesion ability. Moreover, the GelMA-TA hydrogel exhibits good biocompatibility, suggesting it has promising potential for the application of regenerative medicine. The in vivo experiments demonstrated that the GelMA-TA hydrogel could be used for the noninvasive wound closure of skin and gastric wounds under tension or wet conditions [49]. 

### 2.4. Static-Electric Force

The principle of electrostatic adhesion is quite simple: it is an attraction between two different materials in contact (e.g., adhesive and substrate), where free electronic or ionic charge groups exist over the surfaces. The process pertains generally to both nanoparticles and large molecules, and it occurs primarily in liquids [50]. The electrostatic forces have a predominant effect on the movement and location of particles, dominating over weaker and shorter-range van der Waals forces since self-assembly via electrostatics can act over distances much greater than a particle diameter [51].

Electrostatic interactions have been widely used in nanoscale assembly. Oyane et al. have been successful in immobilizing acidic proteins in the apatite layer via electrostatic interactions to create third-generation biomaterials [52]. Recently, Lee and Wu assembled a neuron induction system by the layer-by-layer assembly of PLL/PLGA PEM films via electrostatic interactions and promoted a neural stem/progenitor cell adhesion and differentiation into functional neurons [53].

### 2.5. Chemical Bonds

Chemical bonds formed at an interface can greatly participate in the level of adhesion. The short-range forces involved in chemical bonding are quantum mechanical and generally attractive. The typical strength of chemical bonds, including covalent and ionic bonds, is 100–1000 kJ/mol, whereas that of van der Waals interactions and hydrogen bonds does not exceed 50 kJ/mol. The functional reactive moieties that can be used in tissue adhesives is much more extended, including alkyne, alkene, acetal, orthoester, imidoester, carbamate, thiocarbamate, carbamide, aldimine and ketimine, N-acylurea and O-acylisourea, maleimide, disulfide, epoxide, aziridine, carbodiimide, episulfide, ketene, carboxylate, phosphate, alkyl halides, aryl halides, and alkyl sulfonate esters. However, it is difficult to utilize one of these alternative chemistries in real applications because they are potentially dangerous and can induce infection risks and tissue necrosis [54]. 

Currently, commercially available tissue adhesives depend on chemical bonds, while a limited number of reactive groups can be used for tissue adhesive materials. The most popular functional moieties are methacrylates for photopolymerization and NHS-esters or aldehydes for spontaneous crosslinking with amines. However, methacrylate cannot direct the linkage to tissue and needs external irradiation to start the chemical bond formation. NHS-ester chemistry is very popular in recent adhesives research due to its low toxicity and high reactivity toward amines, which are widely present in the tissue.

## 3. Adhesion Measurements

Adhesion measurements include direct and indirect adhesions. Direct adhesion measurements are commonly used methods and provide qualitative or quantitative adhesive characteristics data, although they do not provide physical interpretations of different adhesion measurements. Indirect measurements, such as XPS, ToF-SIMS and contact angle measurements, can combinate direct measurements for comprehensive investigative adhesion phenomena. This section deals with primarily different, commonly used direct adhesion measurements, including tension tests, peel tests, lap and shear tests, and pull-off /pull-out tests.

### 3.1. Tension Test

Tension testing is widely used to measure the strength of adhesives. It is the application of a uniaxial force to measure the performance of a test sample up to the point of yield or breaking, whether it is sharp or gradual. Tensile test results can provide a detailed profile of the force, extension and time, including partial failure, slippage, and sharp and percentage of breaking. For adhesive testing, the data curve of the force vs extension is largely used to determine ultimate tensile strength [55]. 

### 3.2. Peel Tests

**Figure 2:**
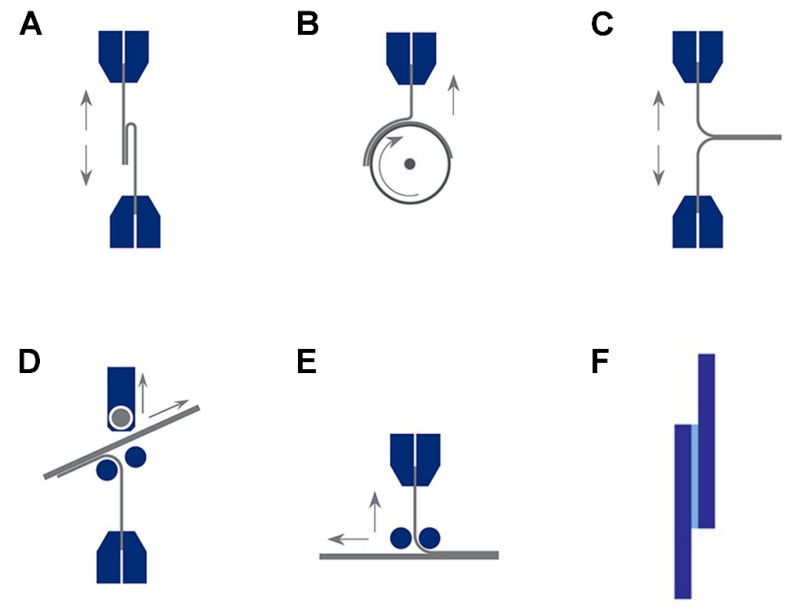
The types of peel tests, including 180 degrees peel (**A**), peel wheel (**B**), T-peel (**C**), Floating roller (115 degrees) (**D**), floating roller or (without rollers) moving table (**E**). The schematic of lap shear tests (**F**). Citing from http://www.mecmesin.com/peel-test-adhesion-testing.

The peel test is a more common quantitative measurement for the resistance of bonded joints to peeling forces [56]. They are chiefly used for the comparative evaluation of adhesives and surface treatment methods because they are very sensitive at discerning differences in the adhesion and cohesion behavior in the adhesive film. Standard test methods tend to prescribe the speed and angle of separation to be repeatable. A tester can ensure the speed is constant, but fixtures are required to maintain the correct angle. Any angle can be used providing it is applied accurately for each test. The figure shows the different types of peel tests, including 80 degree peels, peel wheels, t-peels, floating rollers (115 degrees), or a moving table without rollers (Figure 2A–E).

### 3.3. Lap Shear Tests

The lap shear test is the most commonly used standard test for testing adhesion by pulling bonded layers apart along the plane of the adhesion [49]. The result can be a clean breakaway of the adhesive layer from the substrate, or more likely a breakdown in the cohesion of either the substrate or the adhesive layer, or both (Figure 2F). Following the standard, the overlap corresponds to a width of 25 mm and a length of 12.5 mm. Each end of the sample is held by vice grips and pulled apart at a controlled rate, and the force applied is expressed proportionately to the total adhesive surface area or shear area. A very wide range of tensile lap shear standards has been developed by international and trade organizations.

### 3.4. Pull out/Pull-off Test

Pull-out or pull-off testing is a straightforward tensile test that is performed according to certain standards, but more detail than strength to failure can be obtained [57]. In the case of connectors and terminals with soldered joints or weld nuggets, the test to failure involves a break. In the case of a screw fixture or rivet, or a crimped wire joint, the fixing or the conductor may pull out cleanly. The sample must be held correctly by test fixtures that mimic the end-use and protect the sample; therefore, many specialized fixtures and grips are available. A standard test may require a constant pull speed or pull at a constant hold force, so a motorized or computer-controlled tester is required with a calibrated load cell. Sample elongation and single-strand breaks, as well as slippage within the joint, are factors that will be seen in the test data.

## 4. Adhesives in the Clinic

A wide variety of tissue adhesives have been developed over the past 30 years based on different materials, and they are generally divided into three categories: (1) natural tissue adhesives, (2) synthetic and semisynthetic tissue adhesive, and (3) biomimetic tissue adhesives. Common commercially available tissue adhesives for medical devices are shown in Table 1.

### 4.1. Natural or Biological Adhesives

Tissue adhesives derived from natural polymers, crosslinked via biochemical reactions, offer a more biocompatible alternative to synthetic glues. Examples that are reported in the literature range from protein-based (fibrin-based, collagen-based, gelatin-based) and polysaccharide-based adhesives (chitosan-based, alginate-based, and chondroitin-based) [2,58].

#### 4.1.1. Fibrin-Based Adhesives

The most basic fibrin adhesives consist of two components: thrombin with a small amount of calcium chloride and fibrinogen (with or without factor XIII and fibronectin), which enables the adhesive to mimic the final stages of hemostasis [59,60]. TISSEEL is a commercial fibrin glue, upon mixing human fibrinogen and human thrombin, soluble fibrinogen is transformed into fibrin, forming a rubber-like mass that adheres to the wound surface and achieves hemostasis and sealing or gluing of tissues. TISSEEL mimics the final coagulation cascade step as it has all relevant components to form a clot. It has the longest history and the widest range of applications, including cardiovascular surgery [61,62,63], neurosurgery [64,65,66], plastic surgery [67,68], and spleen and liver lacerations [69,70,71]. However, fibrin-based adhesives show a mechanical strength reduction under wet conditions (e.g., in the presence of a significant amount of blood), and this makes them unsuitable for supporting tissue joints with significant tensile loads to be applied on wet substrates [72,73]. Therefore, they are used in combination with sutures or staples or applied with a carrier sponge to counter brisk bleeding during surgery [74,75].

The risk of disease transmission is a great issue in fibrin-based adhesives since it is derived from human or animal plasma, even when heat inactivation and solvent/detergent extraction methods are used to reduce the risk of viral contamination [76,77]. To avoid the problem of possible contamination, virus-inactivated fibrin sealants were developed. These adhesives offer excellent biocompatibility and low toxicity, while they have a complex preparation procedure, slow curing, and rather poor bonding strength [58].

#### 4.1.2. Collagen-Based Adhesives

Collagen-based adhesives are relatively new and show significant potential [59]. These types of adhesives adsorb blood and coagulation products on its fibers, trapping them in the interstices and effectively adhering to the wound. Moreover, they also induce platelet adhesion and aggregation and activate coagulation factors [78,79]. FloSeal (Sulzer Spine-tech, Anaheim, CA, USA) and Proceed (Fusion Medical Technologies, Mountain View, CA) are two collagen-based adhesives approved for use in the United States, and they are made from a combination of bovine collagen and bovine thrombin and are also relatively inexpensive [59]. 

The collagen-based adhesives, which have a low risk of disease transmission, show very good biocompatibility use records in different medical applications, such as surgical suture materials, hemostatic glue, and wound dressings [80,81,82,83]. Taguchi et al. have prepared collagen adhesives with sufficient bonding strength and low toxicity. The bonding strength is 11 times higher than a fibrin-based glue (Bolheal) but is lower than the cyanoacrylate glue (Dermabond^®^). It requires a long time (10 min) to reach a sufficient bonding strength, which limits its applications in a clinical setting [84,85].

To overcome the abovementioned drawbacks, Baik et al. developed a collagen-based hemostatic adhesive combined with esterified atelocollagen and dopamine and obtained excellent results [86]. However, collagen-based adhesives are not suggested for use for ophthalmologic or urologic surgeries because they can swell with tissue compression [3].

#### 4.1.3. Gelatin-Based Adhesives

Gelatin is irreversibly denatured collagen. It has attracted considerable attention for the application of a variety of soft tissues’ regeneration it can form a gel in situ and serve as an adhesive to bond tissue or seal leaks [87]. Gelatin-resorcinol-formaldehyde (GRF) or gelatin-resorcinol- formaldehyde-glutaraldehyde (GRFG) glue is one of the best-known gelatin tissue adhesives. The first time GRFG was successfully tested was in 1967 in gastrointestinal surgery. It was then used during lung surgery and urinary tract surgery [88,89,90]. GRFG is composed of two solutions: one containing the gelatin and resorcinol solution, the other containing a formaldehyde and glutaraldehyde solution. The disadvantages of the glue include slow degradation, a need to be warmed to 45 °C before application, and toxicity of both formaldehyde and glutaraldehyde. This limits the applicability of this tissue adhesive [89,90,91]. 

Due to the toxicity of both formaldehyde and glutaraldehyde, other crosslinking agents were investigated, such as physical approaches based on self-assembling peptides [92,93], biological means using fibrin sealant [63,68], and biomimetic phenol-mediated crosslinking operations used by insects or mussels [73,94]. Recently, a photochemically crosslinked gelatin-based tissue sealant was developed and demonstrated high adhesive strength, high elasticity, high tensile strength, minimal inflammation, good wound healing and effectively sealed lung, vascular and gastrointestinal defects [95].

#### 4.1.4. Albumin-Based Adhesives

Albumin-based adhesives are fairly similar to the GRFG glue. Glutaraldehyde reacts with the lysine residues in albumin, it works within 20–30 s upon the mixing of the two reagents. BioGlue^®^, containing bovine albumin and glutaraldehyde, forms a relatively strong sealant that has been granted FDA approval for use in acute aortic dissection as well as in cardiac and vascular surgery. The product is best applied to a dry field. Tissue adjacent to the intended application sites should be protected with moist gauze pads to prevent inadvertent contact [3,26]. Drawbacks of this product are mostly the same as for the GRFG glues, which includes induction of cytotoxic effects, tissue compression, myonecrosis, and nerve injury, adhesive embolism, limitation of blood vessel growth, pseudoaneurysm formation, and wound complications. Infections and allergic reactions are also possible. Also, to reduce the risks of glutaraldehyde exposure to operating room personnel, they need to be protected with masks, safety glasses, and gowns.

To reduce the risk of the use of glutaraldehyde, new albumin-based adhesives (ProGel^®^) were developed, which contain human albumin and use NHS end group-functionalized poly (ethylene glycol) as crosslinking agents. ProGel^®^ works within 8 s and can withstand 160 mm Hg of pressure, and it has been approved to seal air leaks on lung tissue after surgery. The disadvantages of ProGel^®^ include that it needs to be refrigerated for storage and has a relatively fast degradation after clinical application.

#### 4.1.5. Chitosan-Based Adhesives

Chitosan is a polysaccharide of b-(1,4)-linked 2-acetamido-2-deoxy-D-glucopyranose and 2-amino-2-deoxy-D-glucopyranose that is produced by partial deacetylation of chitin from crustaceans, fungi, and other non-vegetable organisms. The advantages of chitosan-based adhesives are the biodegradable and biocompatible ability, and low immune responses. Good antimicrobial property is also demonstrated, which is able to inhibit bacterial infections after surgery [9].

Different formulations of chitosan-based adhesives were developed, including fibers, sponges, and hydrogels, and they are mostly used as a wound dressing to accelerate wound healing. Hem Con^TM^ is a chitosan-based adhesive bandage that shows the good hemostatic property in both military and civilian uses [96,97]. Numerous amino groups in chitosan contain positively charged molecules, and they attract red blood cells, boosting the processes of blood clotting and giving a hemostatic property to the adhesive, even in the absence of platelets or coagulation factors. Celox^TM^ is a powder formulation of the chitosan adhesive, and it shows effectively decreased intraoral bleeding time in lingual incisions [98]. The hydrogel forms of chitosan-based adhesives can be achieved by substituting 2% of the amine groups of chitosan with a lactose derivative and adding p-azidobenzoic acid to the substituted chitosan solution to form crosslinks between the polymers via photo crosslink. This adhesive hydrogel successfully seals leakages from pinholes in the aorta and intestine as well as incisions in the trachea.

#### 4.1.6. Dextran-Based Adhesives

Dextran belongs to a polysaccharide that is composed of α-1,6-linked D-glucopyranose straight-chain structure, while branches begin from α-1,3 linkages. It is mostly combined used with PEG or chitosan to form a hydrogel used as a tissue adhesive. Dextran can be oxidized to dextran aldehyde, and adhesion to the tissue occurs via a reaction between the amine groups that exist in the remaining dextran aldehyde moieties and tissue to form imines. This reaction takes less than 1 min to complete. The advantages of these adhesives include low inflammatory response and cytotoxicity, and they are biodegradable [99,100]. The oxidation levels are a key control point for adhesion. If the oxidation level of dextran aldehyde is higher than 60%, the crosslinking reaction is too fast, resulting in insufficient time to bind to the tissue. The desirable oxidation level was found to be approximately 50% [99,100].

A major problem of dextran-based adhesives is the formation of an imine bond in an equilibrium reaction. These adhesives are not very stable in water. They will easily break up and have fast degradation, which does not give enough time to meet the long-term adhesion requirement. To overcome the above-mentioned issue, dextran aldehyde can be replaced by a synthetic analog to enhance the shelf life for commercial use. 

#### 4.1.7. Chondroitin Sulfate-Based Adhesives

Chondroitin is a polysaccharide composed of a chain of alternating N-acetyl-galactosamine and glucuronic acid. This polymer can be sulfated at different positions giving chondroitin sulfate (CS), which is present in cartilage and other tissues. The advantages of CS-based adhesives are biocompatibility and nontoxicity since these polysaccharides already exist in human tissues. However, more functional groups are suggested to modify the CS polymer to obtain better adhesive properties, not only for crosslinking the polymers but also for enhancing binding between the polymer and tissue. Wang et al. developed a multi functionalized CS-based adhesive containing both aldehyde and methacrylate groups. The adhesive was first attached to the cartilage via the aldehyde groups, then poly(ethylene glycol) diacrylate (PEGDA) hydrogel was added and crosslinked with CS, and both in vitro and in vivo studies demonstrated that this adhesive could promote extracellular matrix secretion and cartilage regeneration [101]. 

CS-based adhesives can also be developed by combining CS with a second polymer. Strehin et al. developed a CS-based adhesive through a combinate six-armed starPEG with amine end groups and an NHS-functionalized CS through an amidation reaction. The hydrogel was formed in 49 s at 37 °C, and the adhesion strength was ten times stronger than fibrin glues. Furthermore, the stiffness of the hydrogel can be tuned by changing the pH value [102,103].

### 4.2. Synthetic and Semisynthetic Adhesives

The synthetic and semisynthetic class of adhesives has better adhesion than that of natural adhesion. However, these adhesions present several disadvantages, including low bio-absorption and metabolism, low adherence to wet surfaces and higher cytotoxicity. In addition, it also increases chronic inflammation induced by the release of some of the degradation products. In this section, we will discuss some of the representative synthetic and semisynthetic adhesives, including polycyanoacrylates, polyurethanes, poly (ethylene glycol), polyesters, as well as hyperbranched and dendrimer polymers. 

#### 4.2.1. Polycyanoacrylates

Cyanoacrylates are high reactivity liquid monomers. When applied to tissue, the monomers flow into the cracks and channels of the tissue’s surface, giving a strong bond between the tissue and the glue via covalent bonds between the cyanoacrylate and functional groups in the tissue proteins, such as the primary amines of the lysine side chains. This guarantees adequate adhesive strength for applications on living tissues. Also, they are easy to use and provide an acceptable cosmetic solution, making it suitable for external applications.

Several commercial cyanoacrylate-based tissue adhesives have been approved in the clinic, and Dermabond^®^ is one of the most widely used cyanoacrylate tissue adhesives in cosmetic surgery to avoid using skin sutures as well as in the emergency room to close smaller cuts or reapproximate lacerations that have deep support sutures [104,105,106]. Most importantly, it is mainly applied to superficial or small wounds for children to avoid the trauma of needles. In addition, Dermabond^®^ requires no dressing because it is waterproof.

Dermabond^®^ creates a strong flexible bond, but it is for external use only, because it is able to introduce a serious inflammatory response. Moreover, it is toxic when in contact with noncutaneous tissues [107,108]. Although some internal cyanoacrylate use was reported, applications beyond skin are unwise and there remain some problematic aspects. For example, collagen remodeling was inhibited by inflammatory responses, leading to the delay of wound healing. What’s worse, pancreatic tumor development has been reported [59].

#### 4.2.2. Poly (ethylene glycol)

Poly (ethylene glycol) (PEG) is a hydrophilic biocompatible polymer. In addition, it doesn’t cause a serious immune response. Therefore, it is widely used in the field of medical sciences [109]. There are three main kinds of PEG-based commercial tissue adhesives, including DuraSeal^TM^, FocalSeal^®^ and CoSeal^®^. 

FocalSeal^®^ is approved to stick air leaks by the FDA after lung surgery. A UV light source is used to trigger the photopolymerization to activate the ability of adhesion. In addition to being used in lung surgery, FocalSeal^®^ is also able to repair the ventricular wall [110]. However, the use of FocalSeal^®^ is not widespread due to the limitation of photoactivation, and the generated free radicals during the polymerization may damage healthy tissue. 

DuraSeal^TM^ is a two-component system that contains trilysine (a tetra-amine crosslinker dissolved in sodium borate buffer) and N-hydroxysuccinimide-esters modified four-armed starPEG solution. When the two-component solution was simultaneously sprayed onto the tissue, the amines of trilysine and thiol groups of proteins presented in the tissue quickly reacted with the NHS-esters groups, resulting in a crosslinked network by the formation of amide bonds within seconds. After 4–8 weeks, the adhesive is hydrolyzed, and the degradation products are cleared from the body by renal clearance. However, DuraSeal^TM^ is the uptake of water, and the swell of the hydrogel has the possible oppression of nerves, thus limiting its application.

CoSeal^®^ is also a two-component system that contains a 20% (*w*/*v*) thiol group modified four-armed starPEG polymer solution and a 20% (*w*/*v*) NHS-ester group functionalized four-armed starPEG polymer solution. CoSeal has been approved by the FDA for use in vascular reconstruction. Once the two kinds, if functionalized PEG solutions mixed, the NHS-ester groups rapidly react with the thiol groups leading to the formation of thioester linkage between the two PEG polymers within 3 s and is subsequently hydrolyzed into water-soluble molecules and absorbed after 5–7 days. Compared to DuraSeal^TM^, CoSeal^®^ has a faster degradation in vivo and shows lower stability and relatively weak adhesion to the surrounding tissue. In addition, CoSeal^®^ has a high degree of swelling after application (approximately four times its original size) [3].

**Figure 3:**
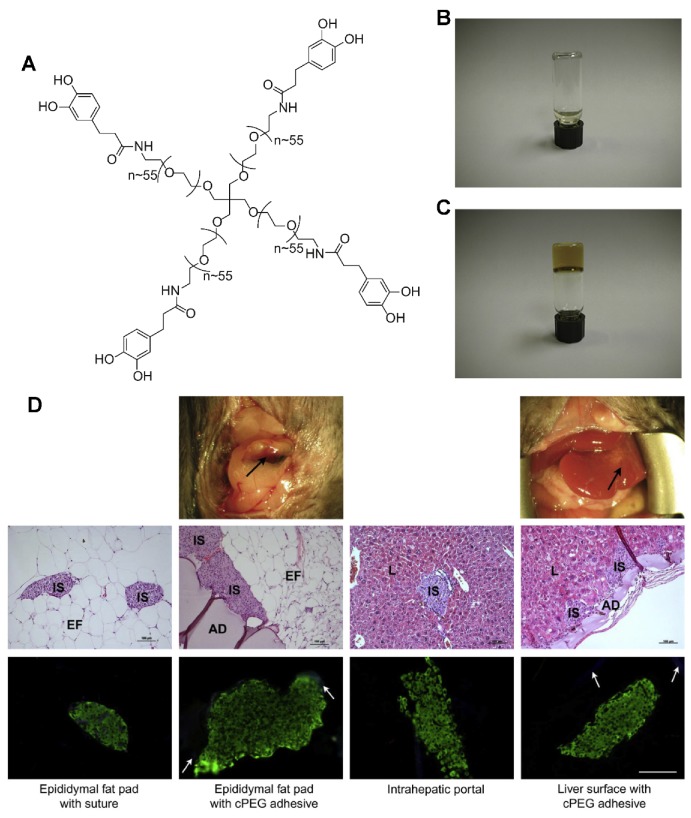
(**A**) Chemical structure of cPEG adhesive precursor. Photographs of precursor solution in phosphate-buffered saline before (**B**) and after (**C**) addition of aqueous sodium periodate solution; gel formation occurred within 20–30 s. (**D**) Analysis of islet graft and cPEG adhesive explants. Top row: photographic images of the site of cPEG adhesive-mediated 150-islet transplantation at the epididymal fat pad and liver surface, immediately before graft explant on day 112. Immobilized islet bolus is visible on the external liver surface. Black arrows, cPEG adhesive. Middle row: representative light micrographs of hematoxylin and eosin (H&E)-stained graft explants. Adhesive, AD; islet, IS; epididymal fat tissue, EF; liver tissue, L. Scale bars: 100 mm. Bottom row: representative fluorescent micrographs of the immunohistochemical triple stain of graft explants. Insulin, green; OX-41 (macrophage marker), blue; CD31 (endothelial cell marker), red. White arrows, non-specific cPEG labeling. All images, scale bar: 100 mm. ^108^ Copyright 2010, Elsevier.

In addition to these commercial PEG adhesive products, Messersmith generated a synthetic adhesive biomaterial for islet transplantation (Figure 3). The adhesive precursor polymer is made of a branched PEG core, whose end groups were derivatizations of catechol, that is a functional group abundantly present in mussel adhesive proteins. Adhesive hydrogels rapidly formed in less than 1 min under oxidizing conditions. After subcutaneous implantation of cPEG adhesive, only minimal acute or chronic inflammatory responses were detected in the interface between supporting tissue and adhesives. In situ formation of cPEG adhesive could efficiently immobilize the transplanted islets at the epididymal fat pad and external liver surfaces, resulting in the recovery of normoglycemic and revascularization of graft. The presented tissue adhesive establishes a new strategy for islet transplantation by the use of synthetic, biologically inspired adhesives [111].

#### 4.2.3. Polyurethanes

The polyurethane-based tissue adhesives show a great promising among the synthetic materials, duo to their perfect thermostability at body temperature without hemolytic behavior and the possibility of being biodegradable and biocompatible [112]. 

TissuGlu^®^ is a polyurethane-based adhesive made of a hyperbranched polymer with isocyanate end groups. Once the adhesive contacts with a wet tissue, it is further crosslinked by hydrolysis of the isocyanate groups into amine groups to form a solid network, in which the residual isocyanate groups react with amine groups to form the solid network via urethane linkages. Additionally, TissuGlu^®^ is able to react with amino groups of proteins contained in biological molecules, the formation of urea linkages enhances the adhesion between the tissues [113]. It has been approved by the CE Mark and is allowed to be sold in the European Union and used for hemostasis, bone fixation, and sealing of vascular grafts, as well as for an abdominoplasty procedure [114].

The downside of TissuGlu^®^ is that the curing time is too long to meet the requirement of surgeries, although it has good adhesion results [112,115]. To overcome the shortcomings of urethane adhesives, Ferreira et al. developed a 2-isocyanatoethylmethacrylate (IEMA) modified PCL, which can be easily crosslinked via UV irradiation [116].

#### 4.2.4. Dendrimers and Hyperbranched Polymers

Hyperbranched polymers and dendrimers consist of highly branched polymers grown from a central core. With different functional groups and specific structures at their periphery, end groups may provide attractive cross-linkable groups for tissue adhesives [117,118]. Carnahan et al. reported dendritic structure adhesives, which consist of polyethylene glycol (PEG) and poly (glycerol-succinic acid) (PGLSA). PGLSA was functionalized with methylmethacrylate (MMA). When the ethyl eosin and the aqueous dendrimer solution were added to the animal eye, an interlaced network between the crosslinked polymer and the tissue formed, and it showed effective results compared to traditional sutures in vitro [119]. However, the major downside of these photo cross-linkable dendrimers is the need for an argon laser to start the polymerization.

To overcome the above-mentioned drawback, a spontaneous crosslinking system consisting of a lysine-based dendrimer with cysteine end groups ([G2]-(Lys)3-(Cys)4) and a range of linear PEG_75_ chains end-functionalized with aldehydes was developed. It was able to close a corneal incision within 3 min at room temperature in an ex vivo model because the 1,2-thiolamine moiety of cysteine is capable of reacting with aldehydes to form a thiazolidine linkage [120]. 

Furthermore, an NHS-activated PEG and hyperbranched poly (ethyleneimine) (PEI) were also developed for tissue adhesives (Adherus). This process consists of the use of two syringes, whereby one syringe contains a PEI solution and the other syringe contains the NHS-activated PEG solution. The adhesive mechanism is the same as the other NHS-contained tissue adhesives, and the hydrogel can be fully resorbed in 90 days [1].

### 4.3. Biomimetic Adhesives

Biomimetic adhesives found mainly in mussels are likely to become the next generations of tissue adhesives for clinic use, since the stable adhesion properties exhibited in a wet environment are identified to meet the requirement of bioadhesives for diverse applications. Mussel- and gecko-inspired adhesives are examples of these adhesives [33,37,38,40,121].

#### 4.3.1. Mussel-Inspired Adhesives

Mussels strongly adhere to different substrates, including metals, glass, plastic and living tissue substances under saline conditions. Moreover, biodegradable, nonimmunogenic, and harmless properties make the mussel inspired adhesives more attractive [40,121,122]. However, uneconomical extraction and unsuccessful large-scale production have limited mussel adhesive application. The natural extraction used to isolate mussel adhesive proteins is a labor intensive and inefficient process, with 1 g of the foot adhesive protein requiring around 10,000 mussels. Further, genetically engineered proteins also failed to obtain large amounts of mussel adhesive protein for several reasons. Fortunately, studies have revealed a higher proportion of DOPA residues in mussel adhesive proteins that are closer to the adhesion interface, and it is responsible for adhesion [122]. Although the exact mechanism of crosslinking is still unclear, it is demonstrated that the oxidation of DOPA to quinone is necessary for crosslink and adhere to surfaces or tissue [123,124].

Cost-intensive extraction and unsuccessful mass production have limited mussel adhesive application. The traditional extraction of mussel adhesive proteins is a labor-intensive and inefficient process: 1 g of the foot adhesive protein requires approximately 10,000 mussels [2,125]. Genetically engineered proteins also failed to obtain large amounts of mussel adhesive protein for several reasons [126]. 

DOPA-functionalized polymers exhibit great potential for the application of tissue adhesives. PEG, PEG-PEO-PEG, and PEG-PCL have been modified with DOPA and demonstrate a higher adhesion than that of the unmodified polymers [127,128,129]. Brubaker et al. generated a mussel-inspired adhesive that consisted of a branched poly (ethylene glycol) (PEG) core with dopamine structure derivatized end groups. In situ adhesive hydrogels were formed in less than 1 min, as the transplanted islets were efficiently immobilized at the epididymal fat pad and the surfaces of external liver, permitting recovery of normoglycemic and revascularization of the graft [130]. Bilic also tested some injectable surgical sealants used for repairing ruptures of the gestational fetal membrane and showed that mussel-mimetic PEG-based tissue adhesives are efficient, nondisruptive, and nontoxic when bonding to fetal membranes [130,131]. 

**Figure 4:**
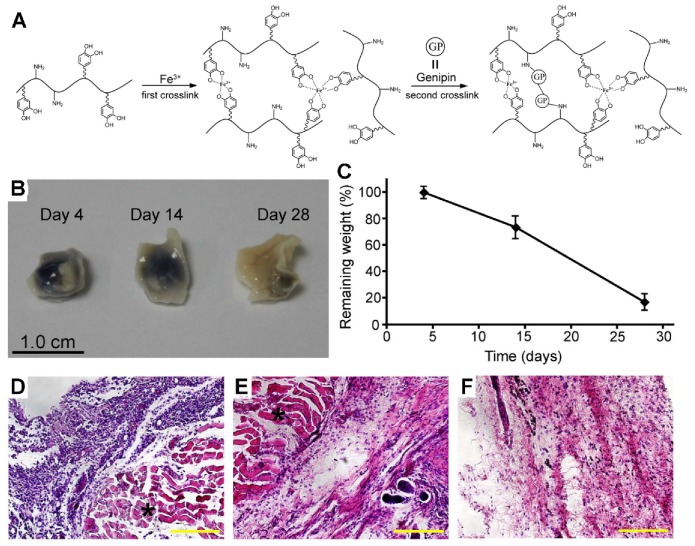
(**A**) Schematic fabrication of DCTA: in one pot, the gelatin–dopamine gluing macromers are first rapidly crosslinked by Fe3+ (first crosslink), at the same time, which are gradually crosslinked with genipin (second crosslink). (**B**) Gross view of the DCTA implants (with murine skins) extracted on day 4, 14, and 28, respectively, after subcutaneous implantation in mice. (**C**) Degradation of DCTA over time after implantation. H&E staining of the tissues surrounding DCTA after 4 (**D**), 14 (**E**), and 28 (**F**) days’ implantation; the DCTA is marked with an asterisk. scale bar:100 μm. ^129^ Copyright 2016, Elsevier.

Wang designed a mussel-inspired, double-crosslinked medical adhesive consisting of dopamine-modified gelatin, a rapid crosslinker (Fe^3+^) and a permanent crosslinker (genipin) (Figure 4). Dopamine is conjugated to the gelatin via chemical reaction inspired by mussel gluing macromer, of which the catechol groups show a strong adhesion on the surface of wet tissue. The double crosslinked networks contain the formation of a catechol–Fe^3+^ complexation and genipin covalent crosslinked gluing macromers. Specifically, the reversible catechol–Fe^3+^ crosslinking promises short-term effectiveness, while the genipin induced covalent crosslinking to execute long-term effectiveness. The ability of wet tissue adhesion of double-crosslinked tissue adhesive was higher than the commercial fibrin glue in a wet porcine skin and cartilage model. Also, good biodegradability, biocompatibility, and elasticity were found in an in vitro study. Moreover, excellent in vivo biocompatibility and biodegradability were confirmed by subcutaneous implantation. This double crosslinked tissue adhesive shows promising potential for internal tissue adhesion and sealing [132].

#### 4.3.2. Gecko-Inspired Adhesives

Gecko-inspired tissue adhesives are another type of biomimetic medical adhesives. Previous studies have shown that millions of nanoscaled fibrils on geckos’ feet act together to create a formidable adhesion through van der Waals forces [33,37]. Based on this knowledge, Geim et al. designed a pillared structure inspired by gecko feet, whereby a mass of the polyimide hairs adhere to the surface, resulting in a highly effective adhesive property [38]. However, the main issues of the gecko-inspired adhesives include quantity production, as well as the endurance of these adhesives and the disappearance of adhesion when submerged in water.

To solve the above-mentioned questions, Lee et al. optimized a gecko mimetic adhesive composing by synthetic polymer-coated an array of nanofabricated polymer pillars to mimic the mussel adhesive protein. It achieved effective adhesive performance in both wet and dry environments for over a thousand contact cycles [40]. Mahdavi et al. also developed a biocompatible and biodegradable gecko-inspired adhesive by changing the size of the nanoscale pillars, including the ratio of the tip to the base diameter and the ratio of the tip diameter to pitch. The pillars dramatically enhanced the adhesion strength on an ex vivo porcine intestine model and an in vivo rat abdominal subfascial model. This gecko-inspired tissue adhesive has promising applications in medical therapies ranging from sealing wounds to the replacement of or assistance with staples or sutures [133].

## 5. Conclusions

Although several tissue adhesives have been developed for medical applications, it is difficult to develop an exclusive tissue adhesive that complies with all of the medical and surgical requirements, including biodegradability, biocompatibility, sustainable hemostasis ability, forceful adhesion to wet tissues, noninterference of the processes of wound healing, simplicity of use, long duration, desirable cosmetic results, cost-effectiveness, and suitability for different human tissues. 

To develop improved tissue adhesives for the closure of wounds of living tissues, a profound knowledge of different ingredients provides a further understanding of the behaviors of tissue adhesives for the future. Each component has a special role to play in terms of the biocompatibility, bonding durability, bonding efficiency, bond strength, and shelf life of the adhesive, and a well-thought-out formulation will play an important role in developing a significant outperformance of new products by introducing some special abilities (hemostasis, antimicrobial activity, and antiinflammation) of different ingredients. Therefore, we believe that the tissue adhesives based on natural polymers, which are crosslinked through biochemical reactions by incorporating a special chemical group, and those biomimetic polymers mimicking tissue adhesives show great potential for the next generations of surgical adhesives.

Altogether, this review clearly explains the mechanisms of adhesion, the methods for adhesion tests, and different kinds of tissue adhesives from both clinical and academic standpoints. Moreover, the development of new tissue adhesives will continue to progress in the coming years, and this review article will provide novel creative design principles for the generation of future tissue adhesives.

## Figures and Tables

**Table 1:** Common commercially available tissue adhesives for medical devices.

Categories	Commerical Product	Manufacturer	Constituents
Natural or biological adhesives	Crosseal	Omrix	Human fibrinogen, human thrombin, human fibronectin, human factor XIII, calcium chloride
	TachoSil	Pharmaceuticals International GmbH	Equine collagen patch, human fibrinogen, human thrombin
	Vitagel	Stryker	Bovine collagen, bovine thrombin, patients own plasma
	GRF	Microval	Gelatin, resorcinol, formaldehyde, glutaraldehyde
	ProGel	NeoMend	Human Serum Albumin, PEG di NHS
	Tisseel	Baxter	Human fibrinogen, human fibronectin, human thrombin, human Factor XIII, bovine aprotinin, calcium chloride
	Artiss	Baxter	Human pooled plasma
	Evicel	Ethicon	Human fibrinogen, human thrombin, human factor XIII, calcium chloride
	CryoSeal	Thermogen	Human fibrinogen, human thrombin, human fibronectin, human Factor XIII, human Factor VIII, human vWF, human thrombin from individual units of plasma
	Hemaseel	Haemacure Corp.	Human fibrinogen, human fibronectin, human factor XIII, bovine thrombin, calcium chloride
	BioGlue	CryoLife	Albumin, glutaraldehyde
synthetic polymer-based tissue adhesive	Histoacryl	B. Braun	n-Butyl-2-cyanoacrylate
	Dermabond	Ethicon	2-Octyl-2-cyanoacrylate
	Octylseal	Medline Industries	2-Octyl-2-cyanoacrylate
	Surgiseal	Adhezion Biomedical	2-Octyl-2-cyanoacrylate
	Omnex	Ethicon	n-Octyl-2-cyanoacrylate/butyl lactoyl-2-cyano acrylate
	Indermil	Henkel	n-Butyl-2-cyanoacrylate
	Liquiband	Advanced Medical Solutions	n-Butyl-2-cyanoacrylate
	Histoacryl Histoactryl Blue	Tissueseal	n-Butyl-2-cyanoacrylate
	Glubran Glubran2	GEM Italy	n-Butyl-2-cyanoacrylate/methacryloxysulpholane
	IFABond	IFA medical	N-Hexyl-2-cyanoacrylate
	TissuGlu	Cohera medical	Lysine di/tri isocyanate-PEG prepolymers
	HemCon	Bandage Pro	Chitosan
	Actamax	Actamax Surgical Material LLC	Dextran aldehyde, 8-arm PEG amine MW 10,000 functionalized with tris(2-aminoethyl)amine
	FocalSeal-L	Focal Inc.	Photopolymerizable PEG-co-poly(lactic acid)/poly(trimethylene carbonate)
	DuraSeal	Covidien	Tetra-NHS-derivatized PEG and trilysine
	CoSeal	Cohesion Technologies	Tetra-NHS-derivatized PEG and tetra-thiol-derivatized PEG
	SprayGel	Covidien	Tetra-NHS-derivatized PEG and tetra-amine-derivatized PEG
	TissuePatch	TissueMed	poly-((N-vinylpyrrolidone)50-co- (acrylic acid)25-co-(acrylic acid N-hydroxysuccinimide ester)25)
	OcuSeal	Hyperbranch Medical Technology	poly(glycerol succinic acid) and PEG–aldehyde
	Adherus	Hyperbranch	Activated PEG and branched poly(ethylene imine)
